# Dr. Sims, on the Cæsarean Operation

**Published:** 1799-12

**Authors:** John Sims

**Affiliations:** New Bridge Street


### *To the Editors of the Medical and Phyfical Journal.*

GENTLEMEN,*

NOT having read any one of the pamphlets that have been publifhed by Mr. HULL or Mr. SIMMONS, on the fubject of the Cæfarean Operation, I have no intention in what follows to enter the lifts in favour of thefe difputants. But what has been advanced by the latter gentleman, in the laft Number of your ufeful Journal, appears to me to re-

---

* We were under the unpleafant neceffity of omitting this valuable communication in our laft, on account of the great prefs of matter previoufly received.—EDITORS.

quire fome notice, as it tends to overturn an eftablifhed rule of practice, by which our conduct ought perhaps on fome occafions to be regulated.

I agree with him, that the only cafe in which a thought of the Cæfarean operation can be reafonably entertained, is, where the pelvis of a woman is fo diftorted as to prevent the delivery of a child, in any way, through its contracted aperture, and confequently where, if nothing be done, the woman muft die undelivered, and the child of courfe, though known to be alive, muft perifh with her. I am willing too to concede, that from all experience hitherto had in this country, the operation will certainly be fatal; yet ftill the queftion is not brought to the point, as he has ftated it; *whether the mother's life fhall be facrificed to fave her child?* becaufe fuch a queftion neceffarily involves in it, at leaft a tacit acknowledgement, that the mother's life can be faved, if no regard be paid to that of the child; for how can that be faid to be facrificed, which is already allowed to be devoted to certain deftruction? If the queftion be fairly ftated, it amounts to this: *When the mother's life cannot poffibly be preferved beyond an exiftence miferably protracted for a few days, is it allowable to run the rifk of fhortening this period for the fake of preferving the life of a child, which muft otherwife perifh with her?* Perhaps, when the queftion is thus put, Mr. SIMMONS will not fo *readily* anticipate an anfwer in the negative. It is not improbable that Eliz. Thompfon, the poor woman on whom the operation was performed at Manchefter, furvived as long as fhe would have done, if fhe had been permitted to perifh undelivered, and fuffered infinitely lefs both in body and mind. Human imagination can hardly conceive any thing more dreadful than the diftreffing anxiety of a woman in the pangs of labour, without hope of delivery: the few hours of her exiftence after the birth of her infant muft have been comparatively happy.

What Mr. SIMMONS has faid refpecting laws human and divine, is totally irrelevant; none of your readers need be told, that taking away life does not in all cafes conftitute murder; and his whole reafoning on this point, applies with equal force againft deftroying the child, where that may be neceffary, to fave the mother.

Through the goodnefs of Providence, a pelvis fo deformed, that a woman cannot, under any management, be delivered of a living child, is comparatively a rare occurrence; but one diftorted to fuch a degree, as not to admit of delivery, even when no regard is paid to the life of the child, is fo very rare, that I have never, nor I truft never fhall meet with it. But as it is impoffible to fay what may happen, every practitioner practitioner ſhould have in his mind certain principles by which his conduct may be regulated under all circumſtances. It may then, I believe, be laid down as a rule of practice generally followed in this country, that in no caſe ſhall the life of the unborn child be put in competition with that of the mother. The queſtions really occurring are therefore, Is the danger to the mother's life, ſuch as to call for the ſacrifice of that of the child? or, on the other hand, Is there good ground to believe that ſuch ſacrifice will be effectual in preſerving the life of the mother? But not to take up your room unneceſſarily, I beg leave to refer to Dr. DENMAN's excellent Introduction to Midwifery; a book that is, or ought to be, read by every practitioner in this country; where this whole buſineſs is diſcuſſed in ſo clear and comprehenſive a manner, as I ſhould have thought might have precluded all diſpute, and will conclude this part of the ſubject in his words: "I am not willing to accept any other "principle but neceſſity, as a juſtification of the Cæſarean operation; "that is, whenever it is propoſed, there ſhall be no other way or me-"thod, by which the life, either of the mother or child, can poſſibly "be preſerved; and the impoſſibility ſhall be confirmed, not by the "opinion of one, but as many competent judges as can be procured. "I ſhould then conſider this operation juſtified by every principle of "religion, and the laws of civil ſociety, by as deciſive and ſatisfactory "evidence, as any other operation, which we never heſitate to propoſe "or to perform."

Thus far I have proceeded upon the ſuppoſition, that the operation is to be conſidered as certainly fatal to the woman upon whom it is performed; but although it has uniformly proved ſo in the caſes that have occurred in this country,* theſe caſes, about twelve in number, are by no means ſufficient to warrant a concluſion, that there is no poſſibility of a more favourable event. A rupture of the uterus has been generally conſidered to be certainly mortal, and probably has proved ſo in twelve times twelve ſucceſſive caſes; yet we now know that women have repeatedly recovered from this moſt dangerous accident. A wound of the uterus then is not, nor can we conclude from twelve, twenty, or even fifty unſucceſsful caſes, that the Cæſarean operation is, in its nature, neceſſarily mortal. In ſo deplorable a ſituation therefore, even when we ſpeak of the mother only, and ſet aſide all conſideration about the child,

* It has been ſaid to have been twice ſucceſsfully performed in England; but as I have not ſeen any ſatiſfactory evidence of ſuch operation, I forbear to mention theſe caſes.

we we may apply CELSUS's rule, Melius est anceps quam nullum experiri remedium.

Having then, I trust, brought the business fairly to this issue, that as cases have occurred,* and may occur again, which allow of no other possible way of delivery than the Cæsarean operation, and that as this operation affords an opportunity of preserving the child, and though a very remote, yet the only, chance of saving the life of the mother also, therefore this operation may be justifiable and necessary: It remains next to consider, whether the operation has been conducted in the best possible manner? or whether means may not be devised of affording a somewhat better chance of recovery to the unfortunate patient than has been hitherto done?

We can infer a priori, that wounds penetrating the cavity of the heart must necessarily be fatal, but no such reasoning will apply to wounds of the uterus; we must therefore seek for some other cause for the fatal termination of this operation. This perhaps may be found in the consideration that the subjects are generally such as no one would select to try the success of any operation upon; that this too is to be undergone in the time of labour, when the irritability of the constitution is very much increased. These circumstances, joined to the baneful effect of atmospherical air admitted into the cavity of the abdomen, may perhaps be sufficient to account for the general want of success. With regard to the subject on whom the operation is to be performed, all that can be in our power is, to take care that it be executed as early in the labour as possible. But to prevent the admission of air into the cavity of the abdomen, it appears to me something essential may be done which has not, as far as I know, been hitherto attempted. As, for instance, all the parts concerned

* It has not appeared to me necessary to prove that such cases have occurred, because in the paper published in the last Number of the Journal, Mr. Simmons seems to allow this. But the Monthly Reviewers, in their criticism on another publication of his, Art. 8, for September, say, that they coincide with him in opinion, that this operation is now superseded by safer means, alluding to the remarkable case published by Dr. Osborne. Where these safer means can be applied, I perfectly agree with this opinion; but surely, in such a case as that of Eliz. Thompson, where no part of the child could be touched with a finger, even when the whole hand was introduced into the vagina, no one acquainted with such operations can believe, that the greatest skill could, by any known means, have extracted a full-grown child through such a pelvis. I have by me casts of the pelvis of two subjects upon whom the operation was performed in London, a mere inspection of which will lead to the same conclusion.

concerned in the operation might be kept under water of a temperature duly regulated, not only during the performance of it, but until the wounds were healed, the procefs for which would probably go on as well under water as we know it does under a poultice; at the fame time a free outlet would by thefe means be given to the difcharges from the vagina. If this fcheme fhould be thought impracticable, ftill an attempt might be made, though in a lefs perfect way, to exclude the atmofpheric air. When the line of incifion through the integuments fhould be fixed upon, two flips of adhefive plafter, fpread upon thick, foft, and elaftic leather, fuch as doe or buck fkin, of a fufficient breadth to fecure a firm hold of the fkin, might be fixed clofe to this line; and immediately after the operation, thefe ftrips of leather might be neatly fewed together with a glover's needle, and the future covered with wax foftened with oil. Or perhaps it may be thought better to cover a large portion of the abdomen with fuch a plafter, and to make the incifion through the leather and the integuments at the fame time, fewing the edges of the leather together after the operation as before propofed. Nor is it fufficient to guard againft the admiffion of air by the incifion made through the integuments; this will likewife readily find its way up the vagina, and through the wound in the uterus. This would be effectually prevented by the firft fcheme of keeping the parts immerfed in water; or the air might be in a great meafure excluded, by keeping the external parts clofely covered with any oval veffel filled with a foft poultice, that might fit clofe and fit eafy. Wifhing only to give the principle of what is to be done, and to leave the means of executing it to the ingenuity of the operator, thefe hints may fuffice.

JOHN SIMS.

New Bridge Street,

*Oct.* 21, 1799.

